# Genomic characterisation of *Escherichia coli* isolated from poultry at retail through Sink Surveillance in Dhaka, Bangladesh reveals high levels of multi-drug resistance

**DOI:** 10.3389/fmicb.2024.1418476

**Published:** 2024-05-30

**Authors:** Alistair R. Davies, Thomas Chisnall, Shamima Akter, Md. Mohibul Hassan Afrad, Mohammad Sadekuzzaman, Shukes Chandra Badhy, Md. Zakiul Hasan, Md. Taifur Rahman, Richard P. Smith, Roderick M. Card, Eric Brum, Md. Golam Azam Chowdhury

**Affiliations:** ^1^FAO Reference Centre for AMR, Department of Bacteriology, Animal and Plant Health Agency, Addlestone, United Kingdom; ^2^Central Disease Investigation Laboratory (CDIL), Dhaka, Bangladesh; ^3^Emergency Centre for Transboundary Animal Diseases (ECTAD), Food and Agriculture Organization of the United Nations (FAO), Dhaka, Bangladesh; ^4^WOAH Collaborating Centre for Risk Analysis & Modelling, Department of Epidemiological Sciences, Animal and Plant Health Agency, Addlestone, United Kingdom

**Keywords:** antimicrobial resistance, *E. coli*, Bangladesh, poultry, plasmid, prevalence

## Abstract

The surveillance of antimicrobial resistance (AMR) in commensal *Escherichia coli* from livestock at slaughter is widely employed to assess the potential for risk to humans. There is currently a limited understanding of AMR in Bangladesh poultry at retail in live bird markets, with studies focussing solely on phenotypic characterisation of resistance. To address this evidence gap we performed antimicrobial susceptibility testing and whole genome sequencing on *E. coli* obtained from chickens from live bird markets in Dhaka in 2018 (*n* = 38) and 2020 (*n* = 45). *E. coli* were isolated from caeca samples following ISO guidelines and sequenced using short and long read methods. Multidrug resistance was extremely common (*n* = 77) and there was excellent concordance between AMR phenotype and the presence of corresponding AMR genes or mutations. There was considerable genomic diversity, with 43 different sequence types detected. Public health considerations included the high occurrence of resistance to ciprofloxacin (*n* = 75) associated with plasmid-residing *qnrS* or mutations in the *gyrA* and *parC* chromosomal genes; and the detection of a tigecycline resistant isolate harbouring *tet*(X4) on an IncHI1A/B-IncFIA mosaic plasmid. Thirty-nine isolates were resistant to azithromycin and harboured *mphA*, with a significant increase in the incidence of resistance between 2018 and 2020. Although azithromycin is banned for veterinary use in Bangladesh it remains an important treatment option for humans. Interestingly, *mphA* confers high-level resistance to azithromycin and erythromycin, and the latter is commonly used on poultry farms in Bangladesh. Seven isolates were colistin resistant and carried *mcr1*. For two isolates hybrid assemblies revealed that *mcr1* resided on a highly conserved IncHI2 plasmid that had 93% nucleotide identity to a plasmid from the published genome of an *E. coli* isolate of Bangladeshi human origin. Six isolates had resistance to third generation cephalosporins, associated with plasmid-residing *bla*_CTX-M-55_, *bla*_CTX-M-65_, or *bla*_DHA-1_. By employing phenotypic and genomic approaches for AMR surveillance we have provided new insights into the potential for One Health AMR linkages in Bangladesh. Employing similar approaches in human and environmental sectors will help inform the One Health approach to addressing AMR, and generate evidence to support mitigation measures such as improved antimicrobial stewardship.

## Introduction

Antimicrobial resistance (AMR) is one of the most pressing issues of the 21st century. AMR leads to increased morbidity, disease burden, healthcare costs and mortality. It is currently estimated that AMR will lead to 300 million global deaths, an 11% loss of livestock production and ~ $100 trillion in financial loss by 2050 (Murray et al., 2022). Such is the concern, the World Health Organisation (WHO) implemented a Global Action Plan (GAP) in 2015, based on a ‘One Health’ approach, with a plan to reduce the developing threat of AMR at the sources (WHO, 2015). AMR does not just affect humans, as the ‘One Health’ approach recognises the health of humans is interconnected with the health of animals and the environment (CDC, 2022; FAO, WHO and WOAH, 2022).

Bangladesh is tackling the threat of AMR through the implementation of a National Action Plan (NAP) incorporating a ‘One Health’ approach (Alam et al., 2017), with substantial coordination between the human health and food producing agriculture sectors (including livestock, aquaculture and crops) (Ahmed et al., 2022). Bangladesh has a large livestock sector, with 403 million terrestrial animals, accounting for 1.5% of the GDP for the national economy. Farming in Bangladesh is comprised of a mixture of intensive and extensive farms. Poultry is the most important and advanced segment of the livestock sector in Bangladesh mostly used for domestic production. The majority of poultry farms are small-medium scale with some larger commercial farms, where farms are categorised as follows: large commercial (>10,001 birds), medium (1,001–10,000 birds), small (101–1,000 birds). Antimicrobial usage on the farms is largely unregulated and antibiotics can be bought off the shelf without the need of veterinary prescriptions, a review paper found 86% of antimicrobials used in livestock from Bangladesh were non-prescription (Morgan et al., 2011; Netherlands Enterprise Agency, 2020).

A 2016 study by Islam et al., observing 73 broiler farms, found all farms were using antibiotics, 31% for prophylaxis and 8% were using antimicrobials for growth promotion. Over 60% of the farms in that study were using antimicrobials without prescription. Nearly 70% of the antimicrobials identified in that study were fluroquinolones, with enrofloxacin and/or ciprofloxacin being used on 19% of farms (Islam et al., 2016). Both enrofloxacin and ciprofloxacin are classified by the WHO as a high priority critically important antimicrobials (HP-CIA) (WHO, 2019). In 2020 a study by Ahmed et al. looked at *E. coli* from 20 broiler farms. The study used whole genome sequencing to characterise isolates resistant to colistin; a final resort antimicrobial reserved for human medicine. The study found that 25% of 1,200 isolates carried *mcr1* genes, aligning to the widespread usage of colistin as a prophylactic for broiler production (Ahmed et al., 2020). In 2022 colistin was banned for use in broiler production by the Bangladeshi government (Directorate General of Drug Administration, 2022).

*E. coli* is regarded as an important indicator species to characterise the transmission and dissemination of AMR, due to its ability to receive and transfer antimicrobial resistance genes (Anjum et al., 2021). This transfer can be to other *E. coli* strains and other bacterial species. *E. coli* is found in the intestinal tracts of mammalian and poultry livestock as well as on skin, fur, and feathers. There are many different strains of *E. coli* that make up normal gut flora and do not cause illness. There are also several pathogenic strains in poultry which can cause illness, such as avian pathogenic *E. coli*, and other strains that can cause infection in humans, these are commonly caused by enteropathogenic, enterotoxiogenic, enteroinvasive, and enterohemorrhagic *E. coli* (Ievy et al., 2020). Contamination of foodstuffs by *E. coli* is often caused by poor food hygiene practises or poorly prepared/cooked meat.

Between 2016 and 2017, a sink surveillance study was carried out in Bangladesh for Highly Pathogenic Avian Influenza (HPAI) and other emerging zoonotic pathogens at Live Bird Markets (LBMs) (Osmani et al., 2018). The sink surveillance study gave an overall picture of the HPAI prevalence across Dhaka, and its effective sampling strategy was readily replicated and adapted for the purpose of bacterial AMR characterisation. Studies by Sarker et al. (2019) and Mandal et al. (2022) indicate a high level of AMR in *E. coli* from poultry, however, few papers have used whole genome sequencing to characterise the resistance genes present in *E. coli* isolated from LBMs in Bangladesh. In a 2023 systematic review (Islam et al., 2023a) 17 articles were identified looking at AMR in *E. coli* from poultry, some articles used PCR to identify antimicrobial resistance genes. No articles used whole genome sequencing, limiting the number of genes that can be identified.

The purpose of this study was to characterise the antimicrobial resistances and genomic diversity of *E. coli* obtained from poultry at retail in Dhaka, Bangladesh. This study at a point close to consumption aimed to address evidence gaps, including the genetic basis of resistance, and allow assessment of the risk that AMR from food producing animals presents to people.

## Materials and methods

### Study design and isolation of *Escherichia coli*

The sample collection followed was based on the sink surveillance protocol for the identification of highly pathogenic avian influenza, which had started in 2016 and collected samples from 106 of the largest selling chicken LBMs in Dhaka (Osmani et al., 2018). In our study, sampling commenced in March 2018, with 24 of the 106 LBMs being randomly selected for sampling. Two whole caeca were collected from each market, one from a Sonali chicken and the other from a broiler chicken, both of which had been freshly slaughtered for retail (no animals were euthanised specifically for this publication). This was to ensure a representation of the two main types of birds consumed in Bangladesh. Sonali is a crossbreed of Rhode Island Red cocks and Fayoumi hens which is well adapted to Bangladesh’s environmental conditions and can command higher prices at retail. Sample collection was not undertaken in April and May 2020 due the COVID-19 pandemic.

For the isolation of *E. coli*, 1 g of caecal contents from chickens was inoculated in 9 mL Buffered Peptone Water (Thermo Scientific) and then incubated at 37°C overnight. The mixture was plated onto MacConkey agar (Thermo Scientific) and incubated for 18–22 h at 37°C. Lactose-fermenting colonies were sub-cultured onto nutrient agar (Thermo Scientific) for biochemical testing. Oxidase and indole testing was carried out and oxidase negative/indole positive isolates were confirmed as *E. coli*. A single colony was selected and stored on beads.

A representative sub-set of 83 *E. coli* isolates was selected for detailed characterisation by antimicrobial susceptibility testing and whole genome sequencing. The selection was undertaken to ensure that both breed and all sampling months were represented, to support the statistical analysis (see below). These 83 isolates were shipped to the UK on charcoal swabs following IATA guidelines and cultured onto CHROMagar ECC (CHROMagar). Isolates were sub-cultured onto MacConkey No.3 (Thermo Scientific) for confirmation of lactose-fermentation. Putative *E. coli* isolates were biochemically tested using oxidase and indole reagents. MALDI-ToF MS (Bruker, Library version 4.7.373.7) was performed for isolates where oxidase/indole testing was inconclusive.

### Antimicrobial susceptibility testing

Antimicrobial susceptibility testing was performed on the 83 isolates by broth microdilution for Minimum Inhibitory Concentration (MIC) determination using commercial plates (Sensititre™ EU Surveillance *Salmonella/E. coli* EUVSEC3 plate, Thermo Fisher Scientific, 2021), according to manufacturer’s instructions. Briefly, a suspension of each isolate was adjusted to a density of 0.5 McFarland in 5 mL demineralised water, then 10 μL of the suspension was transferred to 11 mL of Mueller Hinton broth to obtain a target inoculum density of between 1 × 10^5^ and 1 × 10^6^ CFU/mL. Fifty microlitres was dispensed into each well of the microtitre plate using a Sensititre AIM and incubated aerobically at 35–37°C for 18 to 22 h. Fifteen antimicrobials were tested in this manner (amikacin, ampicillin, azithromycin, cefotaxime, ceftazidime, chloramphenicol, ciprofloxacin, colistin, gentamicin, meropenem, nalidixic acid, sulfamethoxazole, tetracycline, tigecycline and trimethoprim), and MICs were recorded as the lowest concentration preventing visible growth. *E. coli* NCTC 12241 (ATCC 25922) was used as control strain. Susceptibility was assessed using EUCAST ECOFF values (accessed 21/11/2022) (EUCAST, 2022), except for sulfamethoxazole for which the interpretative criteria proposed by the European Food Safety Authority (EFSA) (Amore et al., 2023) were employed as an ECOFF value was not available, as wild type or non-wild type (Schwarz et al., 2010). ECOFFs distinguish microorganisms without (wild type) and with phenotypically detectable acquired resistance mechanisms (non-wild type) to the antimicrobial in question.^1^ In this paper use of the term ‘resistance’, such as multidrug resistance and phenotypic resistance refers to the non-wild type phenotype, which is not necessarily synonymous with clinical resistance (Schwarz et al., 2010; EUCAST, 2022). Isolates resistant to third generation cephalosporins (cefotaxime MIC ≥0.5 mg/L and/or ceftazidime MIC ≥1 mg/L) were additionally tested on the EUVSEC2 microplate (Sensititre®, Trek Diagnostic Systems, East Grinstead, UK) to determine the presumptive phenotype of Extended-Spectrum Beta-Lactamase (ESBL), AmpC and carbapenemase producers (EFSA and ECDC, 2023). The following antibiotics are included in the EUVSEC2 plate: cefepime, cefotaxime, cefotaxime and clavulanic acid, cefoxitin, ceftazidime, ceftazidime and clavulanic acid, ertapenem, imipenem, meropenem, and temocillin. Where isolates presented with resistance to three or more antimicrobial classes they were classified as multidrug resistant (MDR) (Schwarz et al., 2010).

### Whole genome sequencing and analysis

DNA extracts were prepared from overnight Luria-Bertani broth cultures of the 83 isolates with the MagMAX™ CORE extraction kit (Thermo Fisher Scientific, Basingstoke, UK) using the semi-automated KingFisher Flex system (Thermo Fisher Scientific, Basingstoke, UK) according to the manufacturer’s instructions. Extracted DNA was processed for whole genome sequencing using the NextSeq® 500/550 Mid Output Kit v2.5, using NextSeq sequencing reagents. The resulting raw sequences were analysed with the Nullabor 2 pipeline (Seemann et al., 2020), using as reference the published genome *E. coli* K12 (Accession number U00096.2), Spades for genome assembly [version 3.14.1; (Prjibelski et al., 2020)] and Prokka for annotation [version 1.14.6; (Seemann, 2014)]. The presence of genes and point mutations conferring AMR, heavy metal stress genes, and virulence genes were assessed using AMRFinderPlus (Feldgarden et al., 2021). AMR genes and plasmid incompatibility types were identified using APHASeqfinder.^2^ The Sequence Type (ST) was determined with MLST [version 2.19.0; (Seemann, 2022)] using the pubMLST database (Jolley et al., 2018). Core genome SNPs were generated using SNIPPY (Seemann, 2020). *E. coli* serotypes were determined using ECTyper (Petkau et al., 2017). Phylogenetic trees with 200 bootstraps were built using RAX-ML (Stamatakis, 2014) from the core genome SNPs, and annotated using iTOLv5 (Letunic and Bork, 2021).

Three isolates were selected for long read sequencing using Oxford Nanopore Technologies (ONT), based on the presence of AMR genes and plasmids identified through the short read sequencing (see Results). DNA was extracted using the GenFindV3 extraction kit (Beckman Coulter) according to the manufacturer’s instructions. Sample preparation was carried out using the SQK-RBK004 Rapid barcoding kit (ONT) according to manufacturer instructions. Samples were then run-on MinION and MinION flow cell for 72 h. Hybrid assemblies were created using long and short read sequences by Unicycler v2.0 (Wick et al., 2017) to generate closed (fully circularised) plasmids. StarAMR was used to map AMR resistance genes to plasmids (Bharat et al., 2022). Blastn and MOB-suite (Robertson and Nash, 2018; NCBI, 2023) was used to identify similar published plasmids from public database (NCBI, 2023) and BRIG (Alikhan et al., 2011) was used to generate an image that compared published plasmids with those identified in this study. Bakta and the CARD AMR database were used for image annotation (Schwengers et al., 2021; Alcock et al., 2023). Easyfig was also used for comparative analysis using published genomes from NCBI Blast (Sullivan et al., 2011).

The whole genome sequences were deposited in the National Center for Biotechnology Information (NCBI) National Library of Medicine under BioProject accession number PRJNA1100899.

### Statistical analysis

Logistic regression was used to assess for significant (*p*-value <0.05) associations between individual AMR and MDR outcomes and the three available explanatory variables (year, month, and breed). We also analysed whether *E. coli* which were ESBL-producers, AmpC-producers or colistin resistant have associations with ST and breed.

## Results

A total of 315 caeca samples were collected from apparently healthy poultry from live bird markets in the years 2018 (*n* = 180) and 2020 (*n* = 135). *E. coli* was isolated from 217 samples, with 108 isolated in 2018 and 109 in 2019 (Supplementary File S1). The number of samples positive for *E. coli* each month sampled ranged from 11 to 40. A representative collection of 38 isolates from 2018 and 45 isolates from 2020 was selected for detailed characterisation by antimicrobial susceptibility testing and whole genome sequencing (File S1).

### Antimicrobial resistance and carriage of AMR genes

The 83 isolates were assessed for their susceptibilities towards 15 antimicrobials and results are presented in Table 1 and Supplementary File S2. The most common resistances were towards tetracycline (*n* = 78; 94%), ciprofloxacin (*n* = 75; 90%), trimethoprim (*n* = 74, 89%), sulfamethoxazole (*n* = 74; 89%), and ampicillin (*n* = 69; 83%). A noteworthy number of isolates were resistant to critically important antimicrobials (CIAs) as defined by the WHO (2019): azithromycin (*n* = 39; 47%), gentamicin (*n* = 29; 35%), colistin (*n* = 7; 8%) and/or tigecycline (*n* = 2; 2%). Multidrug resistance was common in the panel of isolates (77/83; 93%). All isolates were susceptible to the critically important antimicrobials amikacin and meropenem. Six isolates had resistance to cefotaxime and/or ceftazidime and further susceptibility testing determined that four were Extended-Spectrum Beta-Lactamase (ESBL)-producing *E. coli* and two were AmpC-producers (Supplementary File S2).

**Table 1:** Distribution of minimum inhibitory concentrations from 83 *E. coli* isolates obtained from live bird markets in Dhaka in 2018 and 2020.

Antimicrobial	Antimicrobial concentration test range	ECOFF Values		MIC Range (mg/L)																
		Wild type	Non-Wild type	0.015	0.03	0.06	0.12	0.25	0.5	1	2	4	8	16	32	64	128	256	512	% Resistant
Amikacin	4–128	≤ 8	≥ 16									83								**0%**
Ampicillin	1–32	≤ 8	≥ 16								3	8	3	1	68					**83%**
Azithromycin	2–64	≤ 16	≥ 32								4	12	26	2	6	33				**47%**
Cefotaxime	0.25–4	≤ 0.25	≥ 0.5					77			2	4								**7%**
Ceftazidime	0.25–8	≤ 0.5	≥ 1					76	1	1	2		3							**7%**
Chloramphenicol	9–64	≤ 16	≥ 32										37		1	45				**55%**
Ciprofloxacin	0.015–8	≤ 0.06	≥ 0.12	8			1	8	8	1		3	54							**90%**
Colistin	1–16	≤ 2	≥ 4							76		4	3							**8%**
Gentamicin	0.5–16	≤ 2	≥ 4						42	12	1			28						**34%**
Meropenem	0.03–16	≤ 0.06	≥ 0.12		83															**0%**
Nalidixic Acid	4–64	≤ 8	≥ 16									12	6	6		59				**78%**
Sulfamethoxazole	8–512	≤ 64	≥ 128										6	3					74	**89%**
Tetracycline	2–32	≤ 8	≥ 16								4	1			78					**94%**
Tigecycline	0.25–8	≤ 0.5	≥ 1					75	6	2										**2%**
Trimethoprim	0.25–16	≤ 2	≥ 4					2	6			1		74						**90%**

Susceptibilities have been interpreted using EUCAST ECOFF values (EFSA interpretive criteria for Sulfamethoxazole), which are displayed as vertical black lines. Red fill and blue fill indicate MICs above and below the ECOFF value, respectively.

**Figure 1:**
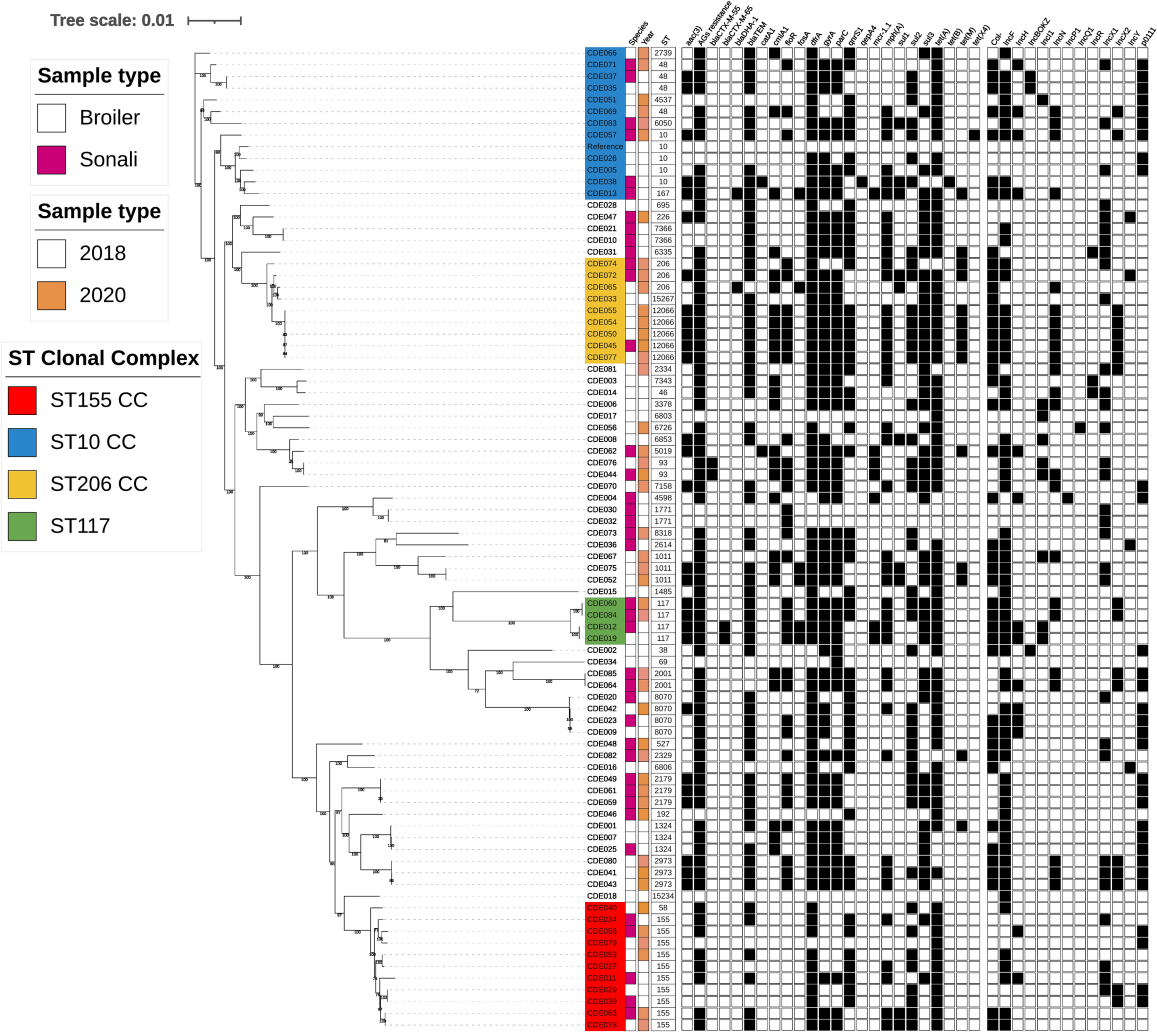
Phylogenetic tree generated from core genome single-nucleotide polymorphisms of *E. coli* isolates obtained from poultry at slaughter in 2018 and 2020. Sequence type, year, species, AMR genes, and plasmid incompatibility types are indicated. Isolate names with colour highlights are discussed in the text and correspond to green (ST117), red (ST155), blue (Clonal Complex 10), and yellow (Clonal Complex 206).

There was very high concordance (>99%) between phenotypic data from MIC and the presence of AMR determinants as determined using genotypic data generated from WGS (Figure 1; Supplementary File S2). All isolates with azithromycin resistance harboured *mph(A)* and all gentamicin resistant isolates contained a variant of *aac3*, with the most predominant being *aac3-lld* (*n* = 25/28). Sixty-nine isolates were resistant to ampicillin, and all carried a variant of the *bla*_TEM_ gene (Supplementary File S2). The two AmpC producers harboured *bla*_DHA-1_ and the ESBL producers harboured *bla*_CTX-M-55_ (*n* = 2) or *bla*_CTX-M-65_ (*n* = 2). Colistin resistance was associated with *mcr1.1* (*n* = 7). Tetracycline resistance was associated with *tet*(A)*, tet*(B) and/or *tet*(M) genes; isolates which carried *tet*(M) (*n* = 14) also carried *tet*(A). Two isolates had a non-wild type tigecycline resistance phenotype; both isolates carried *tet*(A) [but not the variant associated with tigecycline resistance (Radisic et al., 2023)] and one isolate additionally harboured *tet*(X4). Sulfamethoxazole resistance was associated with *sul1*, *sul2* and/or *sul3* genes and trimethoprim resistant isolates harboured *dfrA*. The genes *catA1*, *cmlA1* and *floR* were associated with chloramphenicol resistance; 15 isolates harboured both *cmlA1* and *floR*. Isolates with point mutations in DNA gyrase (*gyrA*) and/or DNA topoisomerase (*parC*), most commonly *gyrA* (D87N), *gyrA* (S83L) and *parC* (S80I) respectively, had high ciprofloxacin MIC values, which exceeded the ECOFF value and also the EUCAST clinical breakpoint of >0.5 mg/L (EUCAST, 2023). Several of these isolates also carried a plasmid mediated quinolone resistance (PMQR) gene: *qnrS* (*n* = 33) or *qepA4* (*n* = 1) (Supplementary File S2). Seventeen isolates had ciprofloxacin MIC values which exceed the ECOFF value but not the clinical breakpoint, and these harboured *qnrS* only. Additional AMR genes conferring resistance to antimicrobials not tested by MIC in this study were present in many isolates (Supplementary File S2).

### Genomic diversity

Considerable genomic diversity was evident in the isolate collection, summarised in the phylogenetic tree (Figure 1). The isolates comprised 44 different sequence types (ST), of which two were newly identified in this study (ST15234 and ST15267). 29 STs were detected only once (Supplementary File S3). The most frequently identified ST was ST155 (highlighted red in Figure 1), detected in both breeds of bird and in each year. AMR gene content and the presence of plasmid incompatibility types differed between ST155 isolates, with only *tet*(A) present in all ten isolates. Five isolates were ST12066, part of clonal complex 206 (highlighted yellow in Figure 1), which were all detected in 2020, in both broiler and Sonali birds. Each harboured the same 19 AMR genes and four plasmid incompatibility types. These isolates were multidrug resistant and possessed AMR genes conferring resistance to eight antimicrobial classes. There was high sequence identity between the ST12066 isolates, with <10 single nucleotide polymorphisms (SNPs) difference between them, therefore meeting proposed relatedness threshold criteria to be considered clones (Schurch et al., 2018).

Four isolates were ST117 (highlighted in green in Figure 1) and these could be grouped into two clones based on sequence identity. One clone comprised the 2018 isolates CDE012 (Sonali) and CDE019 (broiler) which harboured four plasmid incompatibility types and 13 AMR genes, including *bla*_CTX-M-65_ and *mcr1.1*. The two 2020 isolates CDE060 and CDE084 (both from Sonali birds) comprised a different clone carrying 12 AMR genes and four plasmid incompatibility types.

Several sequence types from the ST10 clonal complex were detected (highlighted in blue in Figure 1). ST10 isolates were identified in 2018 and 2020, in both broiler and Sonali chickens, but sequence identity exceeded the proposed threshold to indicate a clonal relationship. The ST10 isolate CDE057 from a Sonali bird carried *tet*(X4), which confers resistance to tetracycline and tigecycline, as well as 10 other AMR genes and six plasmid incompatibility types. Considered as group these four ST10 isolates harboured from 5 to 12 AMR genes, and all possessed the *gyrA* (S83L) mutation, *dfrA* and either *tet*(A) or *tet*(B). Isolate CDE013 resided in the same sub-clade as the ST10 isolates (Figure 1) and was ST167, a member of the ST10 clonal complex. CDE013 had an AmpC resistance phenotype and harboured *bla*_DHA-1_, the colistin resistance gene *mcr1.1* and 13 other AMR genes. Four ST48 isolates (ST10 clonal complex) were also detected. The *E. coli* serotypes were determined using an *in silico* detection method (ECTyper), a range of serovars were identified but sequence type was used to analyse genomic diversity, and no further analysis of serovar was conducted.

### Presence of plasmids harbouring multiple antimicrobial resistance genes

To further explore the diversity of AMR genes and assess whether they resided on plasmids we selected three isolates for long read sequencing as exemplars harbouring resistance to critically important antimicrobials: CDE012 (*mcr1.1*; *bla*_CTX-M-65_), CDE013 (*mcr1.1*; *bla*_DHA-1_), and CDE057 [*tet*(X4)]. Plasmids were present in all three isolates and every plasmid was fully circularised using the hybrid assembly approach (Table 2). Nine of the ten plasmids identified harboured AMR genes, and of these seven had an AMR gene content which would confer resistance to three or more antimicrobials, and hence an MDR phenotype.

**Table 2:** Plasmids identified in the MDR isolates CDE012, CDE013 and CDE057.

Isolate	Plasmid ID	Plasmid size (bp)	Plasmid Incompatibility type	AMR genes located on plasmid
CDE012	pCDE012-1	241,725	IncHI2, IncHI2A	*aadA1, aadA2, aph(3″)-Ib, aph(3′)-Ia, aph(6)-Id, bla*_TEM-1B_*, cmlA1, dfrA14, floR, **mcr-1.1**, mph(A), sul3, tet* (A)
	pCDE012-2	117,899	IncI	*aac(3)-IV, **bla***_CTX-M-65_ *, bla*_TEM-1B_*, fosA3*
	pCDE012-3	106,361	IncFIB	*aadA5, dfrA17*
CDE013	pCDE013-1	234,667	IncHI2, IncHI2A	*aadA1, aadA2, cmlA1, dfrA14, **mcr-1.1**, mph(A), sul3, tet*(A), *tet*(M)
	pCDE013-2	129,417	IncFIB	*aac(3)-Iid, bla*_TEM-1B_ *, mph(A)*
	pCDE013-3	63,856	IncN, IncX1	*aph(3′)-Iia, **bla***_DHA-1_*, fosA4, sul1*
CDE057	pCDE057-1	217,549	IncFIA, IncHI1A, IncHI1B	*dfrA14, floR,* ***tet*(X4)**
	pCDE057-2	153,061	p0111	*aac(3)-Iid, aph(3′)-Ia, bla*_TEM-1B_*, dfrA14, qnrS1, sul2, tet*(A)
	pCDE057-3	111,908	IncFIB	*-*
	pCDE057-4	32,570	IncN, IncX2	*mph(A), qnrS1, tet*(A)

Resistance genes in bold are highlighting genes of particular interest which are discussed in the text.

**Figure 2:**
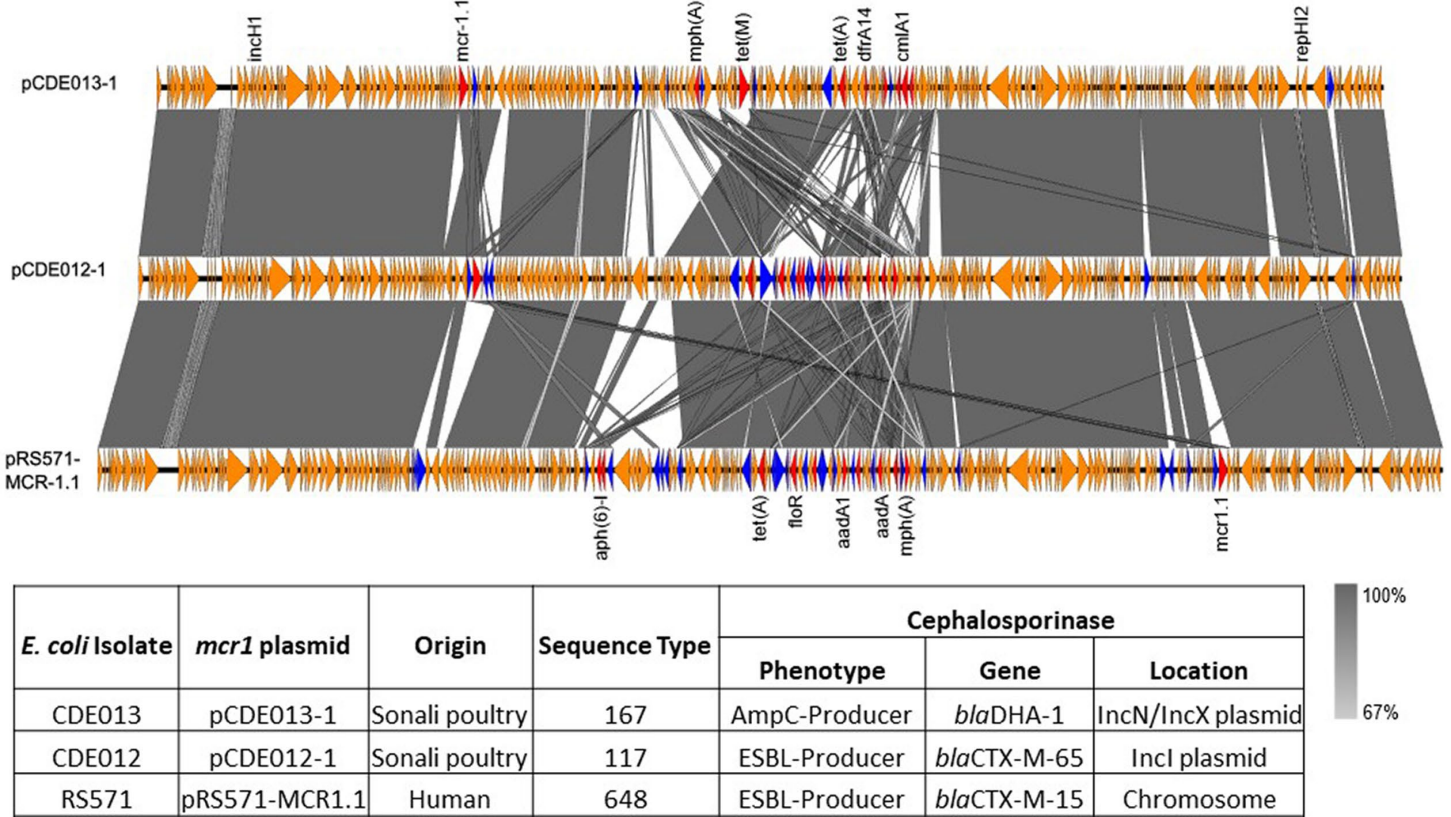
Linear comparison of *mcr1.1* plasmids pCDE013-1 (top), pCDE012-1 (middle) and pRS571-MCR-1.1 (lower). AMR genes are denoted in red, IS family transposons in blue, and other genes in orange. The grey area indicates the blast identities, and the percentage of identity is indicated in the legend. The arrow size is proportional to the gene length. The image was generated using EasyFig with default parameters.

**Figure 3:**
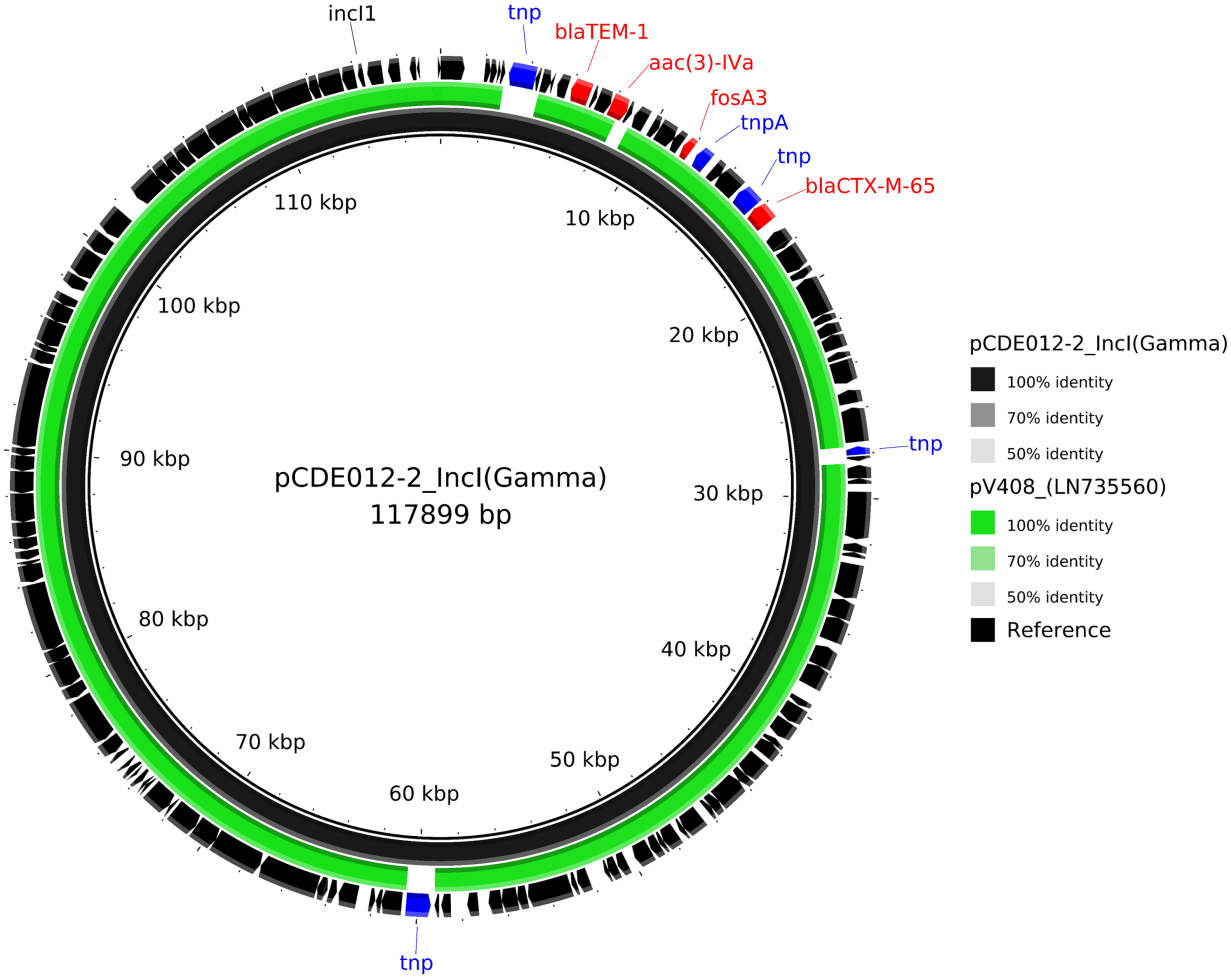
Comparative analysis of the IncI harbouring *bla*_CTX-M-65_ plasmid pCDE012-2 (black inner ring) with the most similar mash neighbour identified using MOB-suite looking at other published plasmids. Each concentric ring displays the nucleotide similarity between pCDE012-2 and the other sequence in the outer ring; LN735560. The shade of colour indicates the BLAST percentage identity. The arrows around the map indicate deduced Coding Sequences and their orientation. AMR genes are highlighted in red, and transposons highlighted in blue. The image was generated using BRIG.

For isolate CDE012, the *mcr1.1* gene resided on a 234,667 bp IncHI2 plasmid (pCDE012-1) together with 12 additional AMR genes (Table 2). Plasmid pCDE012-1 had high sequence identity (>99%) with several published plasmids (Supplementary File S4), including pRS571-MCR-1.1 which was obtained from an *E. coli* reported as isolated from ‘human normal flora’ in Bangladesh in 2018 (accession number CP034390). These two plasmids of Bangladesh origin shared a substantial degree of sequence identity (>99%) and a largely identical gene synteny, however, the *mcr1.1* gene was present in a different location in each plasmid (Figure 2). In both plasmids the *mcr1.1* region was flanked by IS30 family transposons, and it is probable that *mcr1.1* has inserted into these two very similar plasmids at different locations, following separate recombination events. Isolate CDE012 also harboured a 117,899 bp IncI plasmid (pCDE012-2) on which resided *bla*_CTX-M-65_*, fosA3* (conferring fosfomycin resistance), *aac(3)-IV*, and *bla*_TEM-1B_ (Table 2). This plasmid had high sequence identity (>99%) with many published plasmids harbouring the same AMR genes, which were present in *E. coli* from poultry and human sources in various regions such as China, South Korea, and Bolivia (Figure 3). Similarly, plasmid pCDE012-3 had high sequence identity (>99%) to published plasmids from *E. coli* (Supplementary File S4).

**Figure 4:**
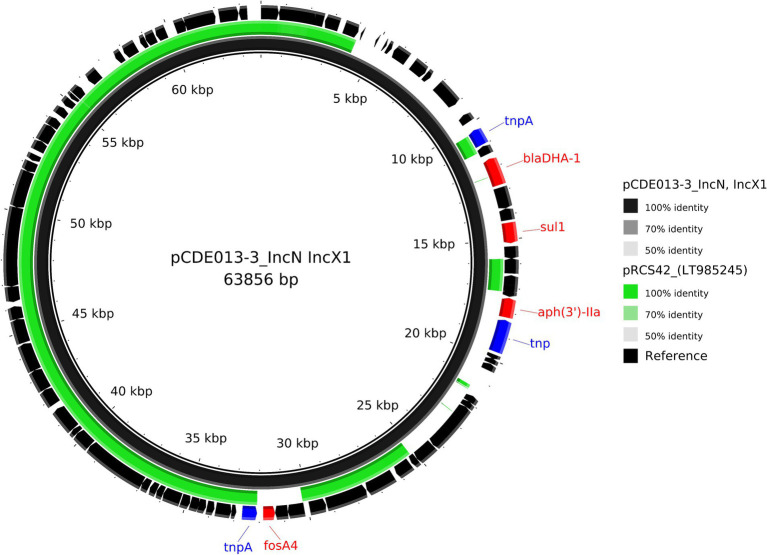
Comparative analysis of the IncN/IncX1 hybrid plasmid pCDE013-3 harbouring *bla*_DHA-1_ (black inner ring) with other published plasmid identified using MOB-suites nearest mash neighbour; LT985245. Each concentric ring displays the nucleotide similarity between pCDE012-2 and the other sequence in the outer ring. The shade of colour indicates the BLAST percentage identity. The arrows around the map indicate deduced Coding Sequences and their orientation. AMR genes are highlighted in red, and transposons highlighted in blue. The image was generated using BRIG.

Isolate CDE013 harboured three plasmids. The *bla*_DHA-1_ gene (conferring the AmpC phenotype) was present on plasmid pCDE013-3, a 63,856 bp IncN/IncX1 hybrid plasmid that also carried *aph(3′)-IIa*, *fosA4*, and *sul1* (Table 2). pCDE013-3 had a backbone region of approximately 40Kb with high sequence identify to published plasmids from *E. coli*, *Salmonella*, and *Klebsiella*, however, the region containing *bla*_DHA-1_, *aph (3′)-Iia*, and *sul1* was not in the backbone region (Figure 4). The most similar plasmids to pCDE013-3 identified by Blast were from *Salmonella* serovar London, however, these plasmids did not contain *bla*_DHA-1_ or *fosA4*. Plasmid pCDE013-2 (harbouring three AMR genes) was similar to previously described plasmids, with high sequence identity and conserved gene synteny (File S4). Plasmid pCDE013-1 was IncHI2 with *mcr1.*1 and eight other AMR genes, and shared extensive gene synteny and nucleotide identity with pCDE012-1 (96% identity) and pRS571-MCR-1.1 (93% identity) (Figure 2), suggesting a highly conserved *mcr1*-containing plasmid is circulating in Bangladesh.

**Figure 5:**
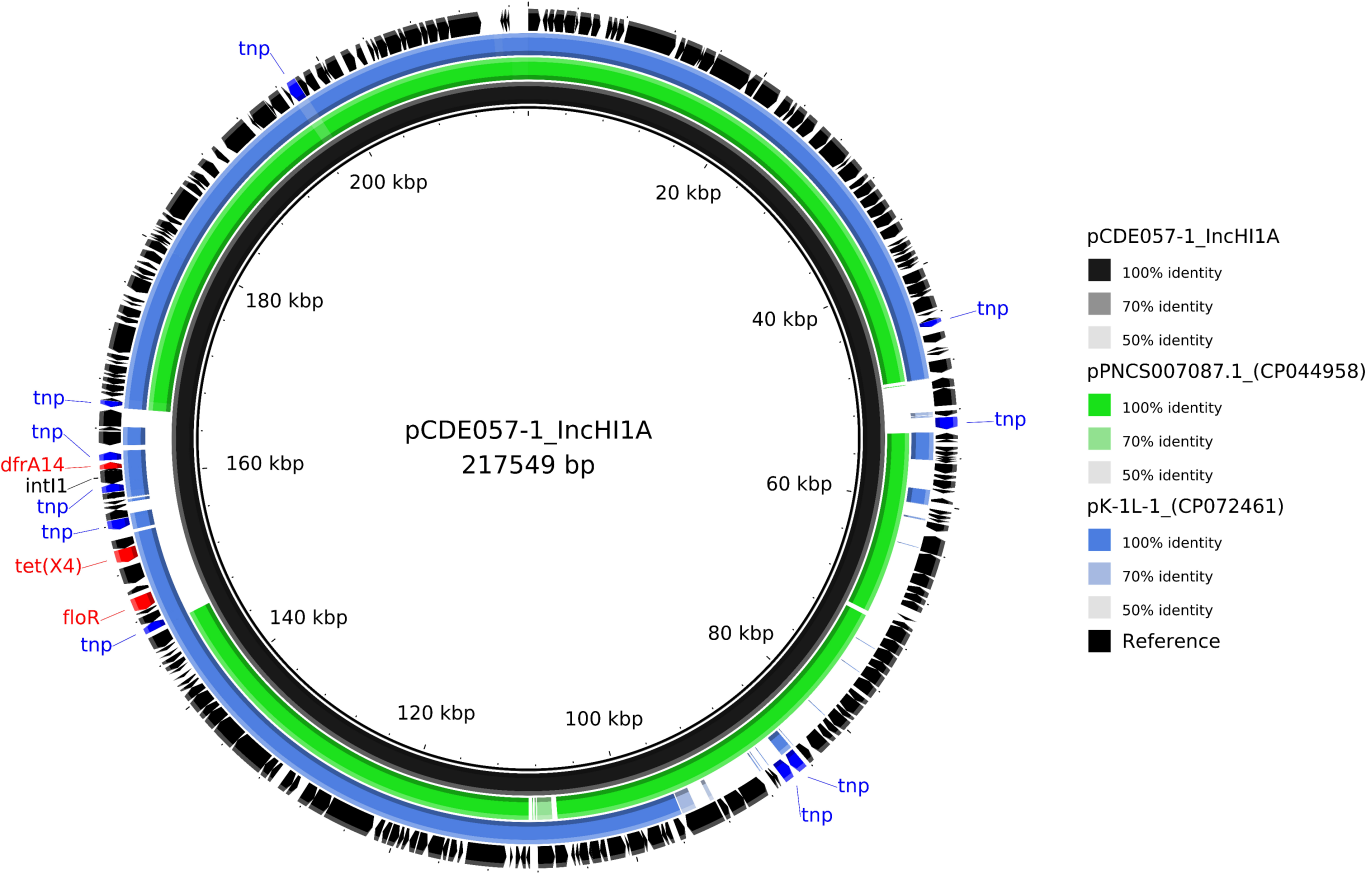
Comparative analysis of the IncHI1A hybrid plasmid pCDE057-1 harbouring *tet*(X4) (black inner ring) with other published plasmids present in *Salmonella* and *K. pneumoniae* isolates (accession numbers from inner to outer ring: CP044958 and CP072461). Each concentric ring displays the nucleotide similarity between pCDE057-1 and the other sequences in the outer rings. The most similar plasmid in green, is missing a large area of homology where the AMR genes reside on plasmid pCDE057-1, hence the comparison with the second plasmid in blue, where the AMR gene region shares highest homology, but the rest of the plasmid shares a lower nucleotide identity. The shade of colour indicates the BLAST percentage identity. The arrows around the map indicate deduced Coding Sequences and their orientation. AMR genes are highlighted in red, and transposons highlighted in blue. The image was generated using BRIG.

For isolate CDE057, the tigecycline resistance gene *tet*(X4) was located on a 217,549 bp plasmid (pCDE057-1) that also carried *drfA14* and *floR* (Table 2). Comparative analysis with published plasmids revealed a ~ 190Kb region with >98% identity to published plasmid from a *Salmonella* monophasic Typhimurium found in Canada, which did not contain any of the resistance genes identified on this plasmid including *tet*(X4) (CP044958). The remaining ~20Kb region containing the AMR genes had the highest homology (>80%) to an IncHI1 plasmid identified in a *Klebsiella pneumoniae* from China (CP072461) (Figure 5). Plasmids pCDE057-2 and pCDE057-4 had <70% sequence identity to published genomes from NCBI and may represent newly described and/or mosaic plasmids; whereas pCDE057-3 was highly similar to previously published genomes but carried no AMR genes (File S4).

### Virulence, disinfectant and heavy metal tolerance genes

The whole genome sequences were also examined for virulence, disinfectant resistance, and heavy metal tolerance genes (Supplementary File S2). All isolates were negative for the *stx* gene, present in Shiga toxin producing *E. coli* (STEC) which can cause serious life-threatening conditions in humans (Melton-Celsa, 2014). One isolate harboured the intimin gene *eae*, a virulence factor associated with the locus of enterocyte effacement (Stevens and Frankel, 2014).

### Statistical analysis

The results of the statistical analyses indicated there was a significantly greater risk of azithromycin (odds ratio (OR) = 3.25, *p*-value 0.011), chloramphenicol (OR 2.75, *p*-value 0.027) and gentamicin (OR 3.88, *p*-value 0.008) resistance in year 2020 when compared to 2018. The month analysis was difficult as many months either had all positives or all negatives and so the analyses predicted success or failure perfectly. However, there was a significantly greater risk of ampicillin resistance in October (OR 9.33 *p*-value 0.038) when compared against the baseline of September, and a significantly lower risk of gentamicin resistance in June (OR 0.07 *p*-value 0.028) when compared against the baseline of January. Due to the study design, however, these associations may be biassed by which year or markets were sampled in those months. The analysis detected no significant associations with bird type.

Similarly, when considering resistances, no significant associations with bird type were detected for the colistin resistance, ESBL, or AmpC outcomes. There were seven *mcr-1.1* isolates, and these had a significantly greater number of resistance genes detected than the other isolates (mean 17.9 compared to 10.4, OR 1.51, *p*-value 0.005). There were four ESBL isolates, and these had a significantly greater number of resistance genes detected than the other isolates (mean 19.5 compared to 10.6, OR 1.96, *p*-value 0.019). The two AmpC isolates had a greater number of resistance genes detected than the other isolates (mean 18.5 compared to 10.9) but this was only approaching significance (OR 1.51, *p*-value 0.094). These three outcomes were not significantly associated with ST or breed.

## Discussion

In this study we assessed the burden of AMR in indicator *E. coli* in poultry at retail in live bird markets in Dhaka, Bangladesh in 2018 and 2020. A representative panel of 83 isolates, from the total of 217 obtained, was selected for detailed characterisation by antimicrobial susceptibility testing using the gold standard broth microdilution and WGS. The use of WGS provided detailed insights into the genetic determinants responsible for the resistance phenotypes observed and showed that resistance is present in a wide diversity of bacterial phylogenies in both broiler and Sonali breeds. Importantly the bacterial isolates did not harbour an *stx* virulence gene, associated with Shiga-toxin and serious life-threatening conditions in humans. Through the use of a hybrid assembly approach, we have demonstrated that many resistances resided on plasmids, which would facilitate the potential for dissemination of resistance. An example from this study is the detection of a highly conserved IncHI2 plasmid carrying *mcr1* and additional AMR genes that was present in *E. coli* isolates CDE012 and CDE013, which are of different STs (i.e., separate phylogenetic lineages). Isolate CDE012 was ST117 and carried an IncI plasmid with *bla*_CTX-M-65_, and was hence an ESBL-producer. Isolate CDE013 was ST167 and carried an IncN/IncX1 plasmid with *bla*_DHA-1_, and hence an AmpC-producer. These data suggest that both isolates have acquired the IncHI2 plasmid independently, demonstrating the potential for AMR dissemination via plasmids, and highlights how accumulation of AMR plasmids can reduce treatment options. Furthermore, database screening showed that the IncHI2 plasmid was also present in an *E. coli* of human origin in Bangladesh from the same year as the poultry isolates. These findings provide an exemplar for the challenge of AMR, where mobile genetic elements are able to move within a bacterial population, and highlight the importance of a One Health approach to AMR surveillance and potential for risk to people via the food chain.

The occurrence of MDR was high at 93%, although similar to recent studies from Bangladesh which have reported MDR at 93 and 100% at LBMs (Azad et al., 2019; Parvin et al., 2022) and 88 and 100% at poultry farms (Rafiq et al., 2022; Ibrahim et al., 2023). Indeed, a recent review paper showed an MDR occurrence of 10–100% in poultry and poultry environment samples in Bangladesh, and of the 14 articles examined nine recorded that 100% of isolates tested had MDR (Islam et al., 2023a). Inappropriate antimicrobial use and inadequate farm biosecurity (which can contribute to disease occurrence and hence antimicrobial use) have been reported for poultry farms in Bangladesh (Imam et al., 2021; Siddiky et al., 2022) and antimicrobial use at poultry farms has been correlated with AMR (Ibrahim et al., 2023). The occurrence of MDR *E. coli* in Bangladesh poultry contrasts with that observed in broilers from other countries, such as the United Kingdom at 27.2% and European Union nations which have a median of 38.3%, although the range is broad at 0.4 to 86.0% (EFSA and ECDC, 2022). To consider the one health context we note that a study of 100 human clinical *E. coli* isolates from Dhaka, Bangladesh, reported 98% MDR (Jain et al., 2021), reflecting the prevalence seen in poultry-derived isolates.

Resistance towards antimicrobials from the WHO AWaRe groups Access or Watch (Zanichelli et al., 2023) was common. Resistance to the Access antibiotics tetracycline, trimethoprim, sulfamethoxazole, and ampicillin was 83–94%; use of these antibiotics has been common in Bangladesh poultry farming (Hassan et al., 2021; Ibrahim et al., 2023). Ninety percent of isolates were resistant to the HP-CIA ciprofloxacin, and this correlated with the presence of mutations in the quinolone resistance determining regions of *gyrA* and/or *parC* (isolates with MIC = > 4 mg/L) and with the presence of plasmid mediated quinolone resistance genes of the *qnrS* family (isolates with MIC 0.25 or 0.5 mg/L). High prevalence of ciprofloxacin resistance in *E. coli* from poultry and humans has been previously reported in Bangladesh (Das et al., 2023; Ibrahim et al., 2023). The use in WGS in this study provided new insights by demonstrating that ciprofloxacin resistance is present in a very wide diversity of *E. coli* STs and lineages (Figure 1), rather than confined to specific clades. The widespread and common use of ciprofloxacin in the Bangladesh poultry sector (Parvin et al., 2022; Ibrahim et al., 2023) will likely have contributed to the high prevalence of resistance, which presents a risk to effective treatment of infection in both poultry and humans.

The high prevalence (47%) of resistance to the Watch group antimicrobial azithromycin was associated with the *mph(A)* gene. In Bangladesh azithromycin has not been commonly used on poultry farms (Ibrahim et al., 2023) and was banned for veterinary use in 2022 (Directorate General of Drug Administration, 2022). However, it remains an important treatment option for invasive *E. coli* infections in humans (Islam et al., 2023a). We note that *mph(A)* confers high level resistance to both erythromycin and azithromycin (Poole et al., 2006) and that erythromycin use in poultry production in Bangladesh is high (Islam et al., 2023a), and therefore consider that this cross-resistance mediated by *mph(A)* may contribute to the high prevalence of azithromycin resistance observed by us and others.

For the ‘Reserve’ antimicrobial colistin, resistance was detected in 7 isolates (8%), mediated by *mcr1* which has been reported as the dominant *mcr* variant in Bangladesh poultry (Ahmed et al., 2020; Ibrahim et al., 2023). In Bangladesh, restrictions on colistin use in the veterinary sector were introduced in 2019 and it has been fully prohibited since 2022 (Directorate General of Drug Administration, 2022). However, colistin has been reported as commonly used by poultry farmers prior to this ban (Ibrahim et al., 2023) and our study was undertaken before the full prohibition.

We detected four ESBL-producing and two AmpC-producing *E. coli*, giving an overall prevalence of 7% for resistance to the ‘Watch’ antimicrobials ceftazidime or cefotaxime. This is similar to the 6% cefotaxime resistance prevalence reported by Ibrahim et al. (2023) but lower than the 22% ESBL prevalence reported for animals in a recent review in Bangladesh (Islam et al., 2023b). The ESBL phenotype was associated with *bla*_CTX-M-55_ (CTX-M group 1) and *bla*_CTX-M-65_ (CTX-M group 9), which have both been reported in ESBL isolates from human clinical samples in Bangladesh (Mazumder et al., 2021). CTX-M group 1 and group 9 genes have been reported in *E. coli* from Bangladesh poultry (Islam et al., 2023a), but detection was by PCR which precluded the definitive assignment of the gene. There has been limited investigation of AmpC-producing *E. coli* in Bangladesh poultry, and here we describe the presence of *bla*_DHA-1_ in two isolates. Distinguishing between ESBL-producing and AmpC-producing *E. coli* is important epidemiologically and also clinically as the latter are not affected by ESBLs inhibitors.

Tigecycline is a ‘Reserve’ antimicrobial and generally only licenced for use in humans, therefore the detection of a tigecycline resistant *E. coli* is significant. Tetracycline has been commonly used in Bangladesh poultry farms (Hassan et al., 2021; Ibrahim et al., 2023) and it has been proposed that the extensive use of tetracycline in livestock has contributed to the dissemination of *tet*(X) variants (Martelli et al., 2022). Tigecycline resistance prevalence in poultry-derived isolates in Bangladesh has been reported at 2 and 27% (Parvin et al., 2022; Ibrahim et al., 2023), but neither study investigated the genetic basis of resistance. Furthermore, tigecycline is known to be light and oxygen sensitive, which can lead to susceptible isolates being falsely described as resistant (Amann et al., 2021). Our genomic approach demonstrated that for one isolate the tigecycline resistance was conferred by *tet*(X4) residing on an IncHI1A/B-IncFIA mosaic plasmid, which had close homology to plasmids detected in *E. coli* obtained from humans. We believe this to be the first description of the *tet*(X4) gene in *E. coli* from Bangladesh poultry. Emergence of plasmid-mediated tigecycline resistance mechanism threatens the use of tigecycline as a last resort antimicrobial against carbapenem resistant, gramme-negative bacterial infections (Zhang et al., 2022). Importantly, in this study we did not identify any resistance to the carbapenem meropenem (Watch group), either phenotypically or genotypically, although carbapenem resistance genes such as *bla*_NDM-1_ and *bla*_KPC-1_ have been identified in human clinical isolates from Bangladesh (Jain et al., 2021).

This study has provided a firm foundation for the phenotypic and genomic surveillance of AMR in poultry at LBMs in Bangladesh. In future we would seek to expand on these finding by including more markets, because for logistical reasons it was not possible to sample all LBMs in Dhaka or sample markets in other cities in Bangladesh. It is also possible that the results of this study may not provide a full representation of the smaller live bird markets in Dhaka, which may obtain birds raised using different farming practises with a concomitant impact on carriage of AMR. Additionally, the sampling strategy was impacted by the COVID-19 pandemic. Expanding the number of isolates tested by MIC and WGS will further contribute.

## Conclusion

In this study we have demonstrated the value of using phenotypic and genomic approaches in a surveillance programme to define antimicrobial susceptibilities and describe in detail the genetic diversity of isolates. This has highlighted important linkages between public and animal health and provided new insights into the potential for transmission between the two sectors in Bangladesh. Ongoing surveillance, susceptibility testing, and genomic characterisation of *E. coli* in poultry at LBMs will increase awareness of resistance prevalence and enable Bangladesh authorities to assess the impact of interventions designed to address the challenge of AMR, such as improved rational antimicrobial use and the prohibition of certain products in the veterinary sector. The statistical analysis determined that there was a significant increase between the two time periods in the incidence of resistance to three antibiotics (azithromycin, chloramphenicol, and gentamicin), which further highlights the need for continued research and surveillance, according to the National Action Plan for Bangladesh (Alam et al., 2017). Employing similar genomic approaches for human and environmental surveillance will facilitate assessment of the One Health impact of AMR and identify novel and emerging AMR genes. Measures to increase the public understanding and awareness of AMR, alongside sustainable implementation of protocols to reduce AMU are required to help mitigate the threat of AMR. For consumers of poultry purchased at live bird markets good hygiene and food preparation practises will help minimise risk of exposure to AMR bacteria.

## Data availability statement

The datasets presented in this study can be found in online repositories. The names of the repository/repositories and accession number(s) can be found at: https://www.ncbi.nlm.nih.gov/, PRJNA1100899.

## Author contributions

AD: Data curation, Formal analysis, Investigation, Methodology, Visualization, Writing – original draft, Writing – review & editing. TC: Data curation, Formal analysis, Investigation, Methodology, Visualization, Writing – review & editing. SA: Data curation, Formal analysis, Investigation, Methodology, Writing – review & editing. MMHA: Investigation, Methodology, Project administration, Writing – review & editing. MS: Investigation, Methodology, Project administration, Writing – review & editing. SB: Investigation, Methodology, Project administration, Writing – review & editing. MH: Writing – review & editing, Data curation, Formal analysis, Investigation, Methodology, Project administration. MR: Data curation, Formal analysis, Investigation, Methodology, Project administration, Writing – review & editing. RS: Formal analysis, Writing – review & editing. RC: Conceptualization, Funding acquisition, Investigation, Methodology, Project administration, Resources, Supervision, Writing – original draft, Writing – review & editing. EB: Conceptualization, Funding acquisition, Supervision, Writing – review & editing. MC: Conceptualization, Funding acquisition, Supervision, Writing – review & editing.

## Footnotes

^1^ https://mic.eucast.org/

^2^ https://github.com/APHA-AMR-VIR/APHASeqFinder
